# Supplementary material for: Gene Set Enrichment Analysis Reveals That Fucoidan Induces Type I IFN Pathways in BMDC
Source: Nutrients. 2022 May 27;14(11):2242. doi: 10.3390/nu14112242 (PMC9182765; doi:10.3390/nu14112242)
Supplement: Supplementary file 1 [file nutrients-14-02242-s001.zip › nutrients-1734173-supplementary.pdf]

Supplementary Materials

Gene set enrichment analysis reveals that Fucoidan induces type I IFN pathways in BMDC

Figure S1.

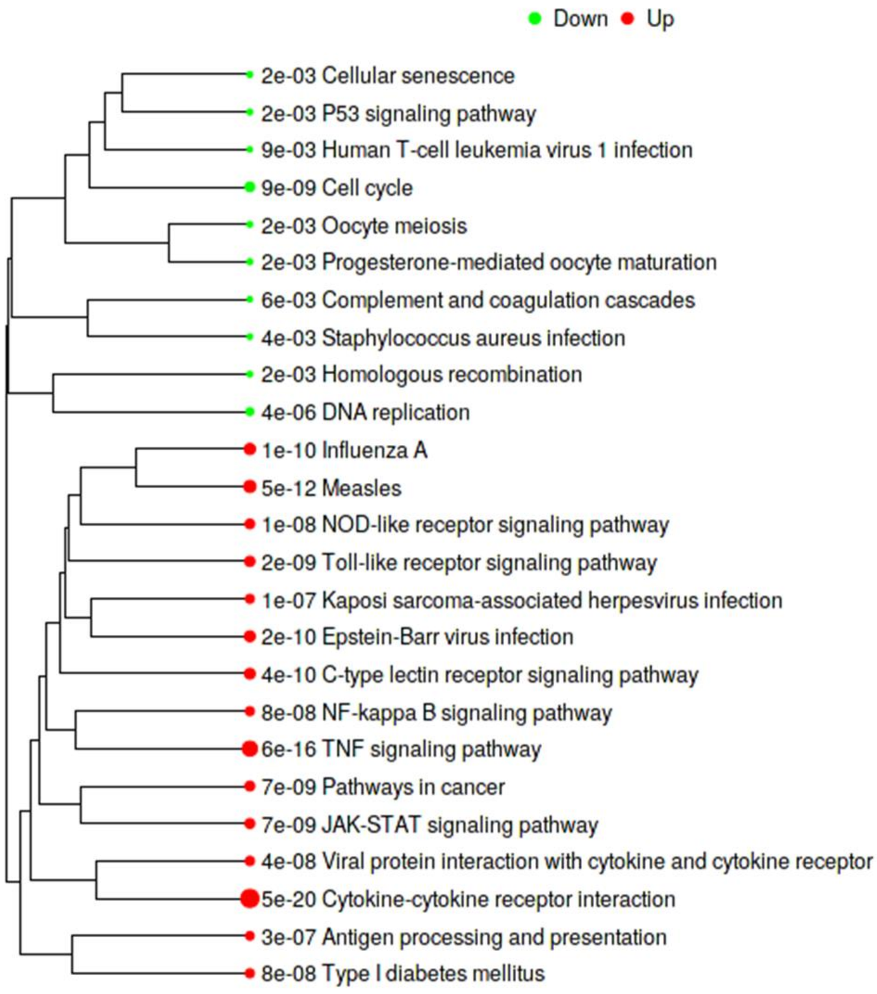

Figure S1. Visualization of the relationships between enriched KEGG pathways in DEGs using hierarchical clustering tree

Table S1. Enriched TF Target gene sets among DEGs

Direction	Pathways	nGenes	adj.Pval
Down	Mcm2 target gene	56	6.70E-44
Regulated	Mcm3 target gene	56	1.10E-43
	Mcm7 target gene	51	6.70E-39
	Trip13 target gene	47	1.60E-37
	Kntc1 target gene	54	2.30E-36
	Foxm1 target gene	37	1.60E-33
	Mcm4 target gene	45	1.60E-33
	Mcm6 target gene	44	2.80E-33
	Espl1 target gene	35	6.10E-31
	Orc2 target gene	20	4.80E-14
	Cenpk target gene	24	2.30E-13
	Timeless target gene	14	2.80E-12
	Hmgb2 target gene	14	5.60E-10
	Rb1 target gene	16	2.10E-07
	Rfc1 target gene	7	7.80E-05
Up	Irf7 target gene	49	5.10E-34
Regulated	Stat1 target gene	100	7.30E-20
	Nfkb1 target gene	54	1.70E-19
	Irf9 target gene	28	3.20E-19
	Irf5 target gene	24	9.10E-17
	Rela target gene	59	3.10E-14
	Irf1 target gene	56	4.50E-13
	Irf8 target gene	18	4.20E-11
	Relb target gene	14	4.90E-10
	Irf6 target gene	14	6.10E-09
	Pml target gene	18	1.80E-08
	Ikbkg target gene	23	1.70E-07
	Irf4 target gene	14	2.20E-07
	Nfkbiz target gene	9	3.40E-07
	Nfkb2 target gene	16	4.00E-07

Figure S2.

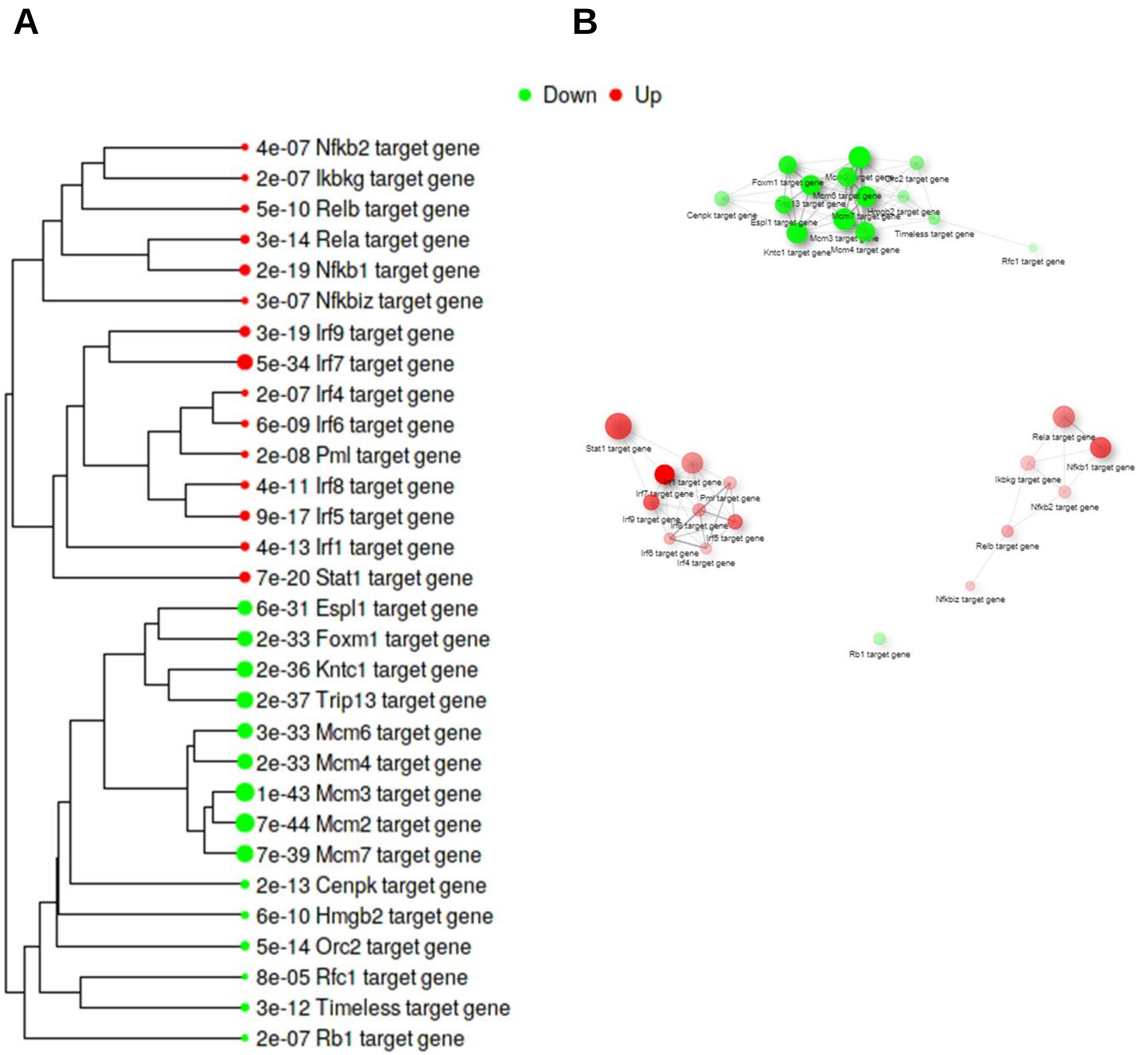

Figure S2. Visualization of the relationships between enriched TF target gene sets in DEGs using (A) tree and (B) network

Table S2. Virus-specific immune pathways enriched in fucoidan-treated BMDCs

NAME	SIZE	NES	NOM p-val	FDR q-val
BLANCO_MELO_COVID19_SARS_COV_2_INFECTION_CALU3_CELLS_UP	277	2.36	0.000	0.000
BLANCO_MELO_RESPIRATORY_SYNCYTIAL_VIRUS_INFECTION_A594_CELLS_UP	242	2.33	0.000	0.000
BLANCO_MELO_HUMAN_PARAINFLUENZA_VIRUS_3_INFECTION_A594_CELLS_UP	165	2.32	0.000	0.000
BLANCO_MELO_COVID19_BRONCHIAL_EPITHELIAL_CELLS_SARS_COV_2_INFECTION_UP	121	2.30	0.000	0.000
BLANCO_MELO_BRONCHIAL_EPITHELIAL_CELLS_INFLUENZA_A_INFECTION_UP	110	2.00	0.000	0.003
WP_SARSCOV2_INNATE_IMMUNITY_EVASION_AND_CELLSPECIFIC_IMMUNE_RESPONSE	62	1.92	0.000	0.008
BLANCO_MELO_COVID19_SARS_COV_2_INFECTION_A594_ACE2_EXPRESSING_CELLS_UP	400	1.92	0.000	0.009
BLANCO_MELO_COVID19_SARS_COV_2_LOW_MOI_INFECTION_A594_ACE2_EXPRESSING_CELLS_UP	119	1.88	0.000	0.014
ZHU_CMV_8_HR_UP	35	1.86	0.000	0.016
WP_SARS_CORONAVIRUS_AND_INNATE_IMMUNITY	27	1.83	0.000	0.022
WP_HOSTPATHOGEN_INTERACTION_OF_HUMAN_CORONA_VIRUSES_INTERFERON_INDUCTION	32	1.82	0.000	0.023
WP_TYPE_I_INTERFERON_INDUCTION_AND_SIGNALING_DURING_SARSCOV2_INFECTION	28	1.82	0.005	0.024
BROWNE_HCMV_INFECTION_4HR_UP	45	1.79	0.005	0.029
VART_KSHV_INFECTION_ANGIOGENIC_MARKERS_UP	157	1.73	0.000	0.041
BLANCO_MELO_COVID19_SARS_COV_2_INFECTION_A594_ACE2_EXPRESSING_CELLS_RUXOLITINIB_UP	342	1.71	0.000	0.049
BLANCO_MELO_COVID19_SARS_COV_2_INFECTION_A594_CELLS_UP	71	1.69	0.000	0.052
BROWNE_HCMV_INFECTION_6HR_UP	56	1.69	0.006	0.053
WP_STING_PATHWAY_IN_KAWASAKILIKE_DISEASE_AND_COVID19	18	1.63	0.007	0.076
KUROZUMI_RESPONSE_TO_ONCOCYTIC_VIRUS	42	1.61	0.006	0.083
DORN_ADENOVIRUS_INFECTION_24HR_DN	38	1.57	0.017	0.098
DORN_ADENOVIRUS_INFECTION_32HR_DN	36	1.56	0.016	0.103
DORN_ADENOVIRUS_INFECTION_48HR_DN	37	1.56	0.016	0.103
DEBIASI_APOPTOSIS_BY_REOVIRUS_INFECTION_UP	258	1.51	0.000	0.122
ZHU_CMV_ALL_UP	100	1.47	0.003	0.145
DORN_ADENOVIRUS_INFECTION_12HR_UP	25	1.41	0.056	0.178
BROWNE_HCMV_INFECTION_12HR_UP	93	1.40	0.009	0.180
BLANCO_MELO_MERS_COV_INFECTION_MCR5_CELLS_DN	24	1.41	0.040	0.180
KUROZUMI_RESPONSE_TO_ONCOCYTIC_VIRUS_AND_CYCLIC_RGD	19	1.34	0.098	0.225
WP_EBOLA_VIRUS_PATHWAY_ON_HOST	103	1.33	0.013	0.229

Table S3. PRR signaling pathways enriched in fucoidan-treated BMDCs

NAME	SIZE	NES	NOM p-val	FDR q-val
KEGG_RIG_I_LIKE_RECEPTOR_SIGNALING_PATHWAY	63	1.76	0.003	0.036
WP_TOLLLIKE_RECEPTOR_SIGNALING_PATHWAY	99	1.74	0.000	0.039
KEGG_TOLL_LIKE_RECEPTOR_SIGNALING_PATHWAY	97	1.73	0.000	0.041
WP_FIBRIN_COMPLEMENT_RECEPTOR_3_SIGNALING_PATHWAY	42	1.70	0.000	0.051
REACTOME_BINDING_AND_UPTAKE_OF_LIGANDS_BY_SCAVENGER_RECEPTORS	36	1.65	0.003	0.071
WP_REGULATION_OF_TOLLLIKE_RECEPTOR_SIGNALING_PATHWAY	137	1.63	0.000	0.076
WP_STING_PATHWAY_IN_KAWASAKILIKE_DISEASE_AND_COVID19	18	1.63	0.007	0.076
KEGG_CYTOSOLIC_DNA_SENSING_PATHWAY	49	1.63	0.006	0.076
WP_NOVEL_INTRACELLULAR_COMPONENTS_OF_RIGILIKE_RECEPTOR_RLR_PATHWAY	57	1.59	0.003	0.088
WP_TLR4_SIGNALING_AND_TOLERANCE	28	1.54	0.018	0.110
KEGG_NOD_LIKE_RECEPTOR_SIGNALING_PATHWAY	53	1.53	0.003	0.111
REACTOME_DECTIN_1_MEDIATED_NONCANONICAL_NF_KB_SIGNALING	60	1.49	0.012	0.131
REACTOME_CLEC7A_DECTIN_1_SIGNALING	97	1.46	0.003	0.151
WP_CYTOSOLIC_DNASENSING_PATHWAY	65	1.44	0.009	0.163
REACTOME_SCAVENGING_BY_CLASS_A_RECEPTORS	18	1.40	0.079	0.180
WP_TOLLLIKE_RECEPTOR_SIGNALING_RELATED_TO_MYD88	32	1.36	0.055	0.207
REACTOME_TOLL_LIKE_RECEPTOR_TLR1_TLR2_CASCADE	100	1.35	0.010	0.217
REACTOME_TOLL_LIKE_RECEPTOR_CASCADES	148	1.30	0.004	0.249
REACTOME_TOLL_LIKE_RECEPTOR_9_TLR9_CASCADE	92	1.30	0.031	0.249

Table S4. Leading edge genes within representative enrichment plots shown in Fig. 4b

(Left)

GENE NAME	RUNNING ES
ADAR	0.023
IRF8	0.057
OAS2	0.085
GBP2	0.115
IRF9	0.146
STAT1	0.173
OAS3	0.201
XAF1	0.224
STAT2	0.249
SOCS3	0.276
IFIT1	0.301
IRF4	0.326
USP18	0.354
ISG15	0.378
RSAD2	0.405
MX2	0.431
IFNAR1	0.458
IRF7	0.484
IFIT2	0.509
IFIT3	0.531
BST2	0.55
IFI35	0.573
SOCS1	0.587
ISG20	0.607
IFITM3	0.61
IFNAR2	0.623
IFNB1	0.633
IRF1	0.648
PSMB8	0.663

(Middle)

GENE NAME	RUNNING ES
NFKBIA	0.068
TRAF6	0.119
NFKB1	0.175
OAS2	0.219
IRF9	0.267
EIF2AK2	0.312
STAT1	0.359
OAS3	0.404
STAT2	0.44
TLR7	0.484
TBK1	0.517
IFNAR1	0.559
TREML4	0.597
IKBKG	0.636
IKBKE	0.633
IFNAR2	0.654
JUN	0.658
MAPK8	0.678
IKBKB	0.701

(Right)

GENE NAME	RUNNING ES
IRF9	0.066
DDX58	0.151
STAT1	0.232
IFIH1	0.306
STAT2	0.378
TBK1	0.442
IFNAR1	0.515
IKBKE	0.53
IFNAR2	0.574
IFNB1	0.616
TLR3	0.659
TICAM1	0.685
JAK1	0.693
TRAF3	0.69
IFNA4	0.712

Figure S3. Leading edge analysis of cluster A1 enriched genes

A

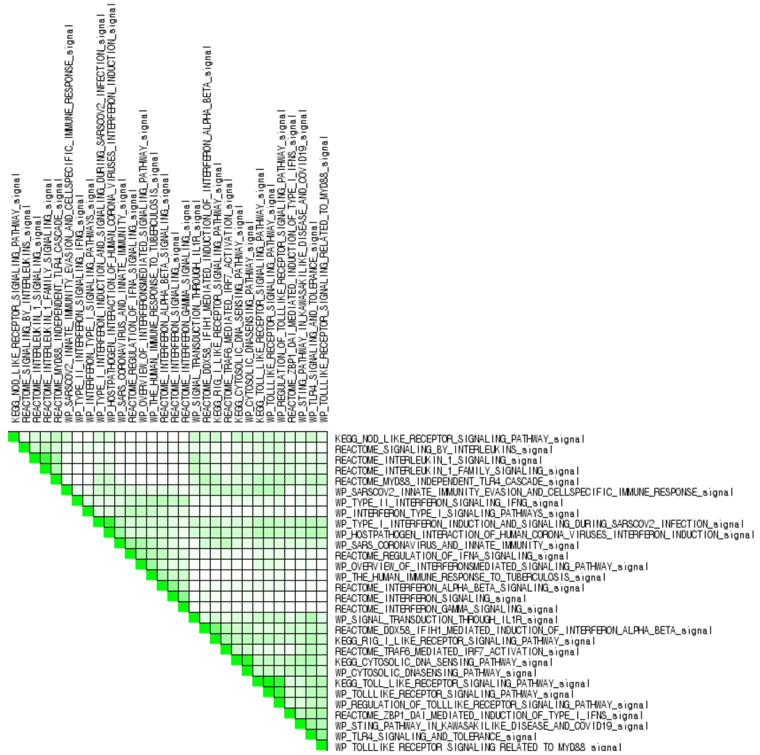

B

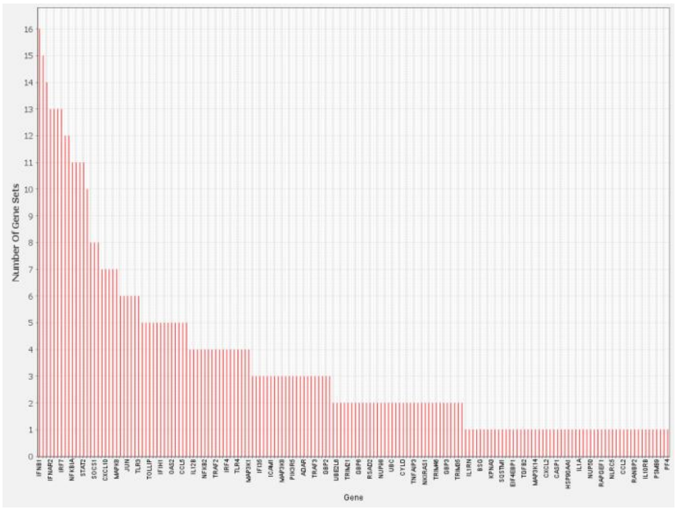

C

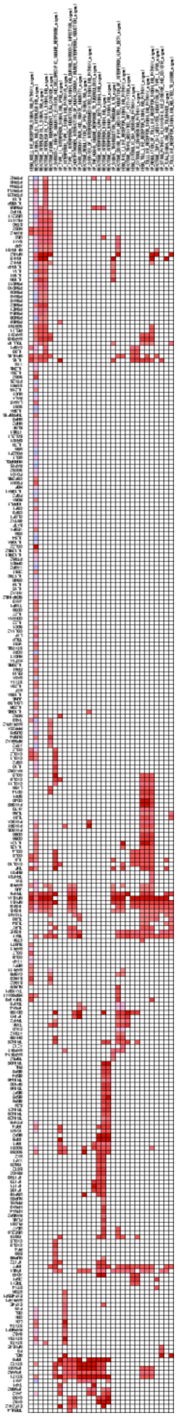

Table S5. GSEA of TF targets in fucoidan vs control BMDCs

NAME	SIZE	NES	NOM p-val	FDR q-val
ICSBP_Q6	239	1.69	0	0.09
IRF2_01	119	1.6	0	0.116
NFKB_Q6_01	228	1.41	0	0.127
NFKAPPAB_01	241	1.41	0	0.135
CREL_01	248	1.46	0.004	0.135
STTTCRNTTT_IRF_Q6	178	1.43	0	0.136
STAT5A_01	236	1.38	0	0.136
ARP1_01	149	1.37	0.008	0.138
NFKB_Q6	242	1.42	0	0.139
SREBP1_Q6	235	1.38	0.005	0.139
GGGNNTTTCC_NFKB_Q6_01	127	1.47	0	0.141
IRF1_01	228	1.39	0.004	0.142
NFAT_Q6	224	1.36	0	0.142
IRF7_01	237	1.54	0	0.143
NFKB_C	249	1.43	0	0.146
IRF_Q6	229	1.36	0.005	0.147
EN1_01	107	1.38	0.009	0.148
E2F_Q6	210	-1.81	0	0.001
E2F1_Q3	221	-1.82	0	0.001
E2F1DP1RB_01	209	-1.78	0	0.001
E2F_Q4	213	-1.79	0	0.001
SGCGSSAAA_E2F1DP2_01	150	-1.83	0	0.001
E2F_02	214	-1.74	0	0.001
E2F_Q3	205	-1.83	0	0.001
E2F4DP2_01	214	-1.73	0	0.001
E2F1_Q4_01	208	-1.77	0	0.001
E2F1DP1_01	214	-1.74	0	0.001
E2F4DP1_01	216	-1.83	0	0.001
E2F_Q4_01	216	-1.75	0	0.001
E2F1DP2_01	214	-1.72	0	0.002
E2F1_Q6	212	-1.83	0	0.002
E2F_Q3_01	214	-1.87	0	0.002
E2F_Q6_01	218	-1.68	0	0.002
E2F1_Q6_01	223	-1.66	0	0.003
E2F_03	229	-1.61	0	0.005
E2F1_Q4	232	-1.61	0	0.005
E2F1_Q3_01	231	-1.54	0	0.015
KTGGYRSGAA_UNKNOWN	70	-1.4	0.016	0.094
NFY_C	218	-1.4	0.001	0.097

Table S6. Leading edge genes within LINDSTEDT DC maturation gene set enri chment plots in Fig. 5a

A

GENE NAME	RUNNING ES
LAD1	0.050
COL14A1	0.091
STAT5A	0.132
ENPP4	0.169
TNFSF4	0.203
LY75	0.233
SYNGR3	0.265
STK39	0.292
CD86	0.320
CD200	0.341
LGALS9	0.349
ATF3	0.372
TNFRSF11B	0.395
SEMA7A	0.416
NID1	0.434
STAT3	0.446
FOXO1	0.467
NFIL3	0.478
FERMT2	0.496
TRAFD1	0.509
SOCS2	0.525
ARID5A	0.538
GTF2E2	0.550
PLAGL1	0.566
S1PR1	0.577
PRKAR2B	0.582
FLT3	0.578
MUC1	0.583
TNFRSF11A	0.583
ROCK2	0.589
JAK1	0.595

B

GENE NAME	RUNNING ES
NR4A3	0.053
NFKBIA	0.087
REL	0.122
NFKB2	0.156
TNIP1	0.190
CD83	0.215
CD80	0.244
MARCKSL1	0.273
NFKB1	0.302
PSME2	0.330
BIRC3	0.356
CYTIP	0.384
TNFAIP3	0.412
DUSP4	0.438
CFLAR	0.464
TRAF1	0.482
TNFRSF9	0.503
SLAMF1	0.522
IL2RA	0.545
IL15RA	0.568
TAP1	0.581
SDC4	0.602
JRK	0.624
ADM	0.644
USP12	0.663
BTG1	0.678
OPTN	0.694
TNFAIP2	0.697
MAP3K14	0.715
PDGFA	0.718
ID2	0.734
PNRC1	0.745
HIVEP1	0.747

C

GENE NAME	RUNNING ES
LAD1	0.050
COL14A1	0.091
STAT5A	0.132
ENPP4	0.169
TNFSF4	0.203
LY75	0.233
SYNGR3	0.265
STK39	0.292
CD86	0.320
CD200	0.341
LGALS9	0.349
ATF3	0.372
TNFRSF11B	0.395
SEMA7A	0.416
NID1	0.434
STAT3	0.446
FOXO1	0.467
NFIL3	0.478
FERMT2	0.496
TRAFD1	0.509
SOCS2	0.525
ARID5A	0.538
GTF2E2	0.550
PLAGL1	0.566
S1PR1	0.577
PRKAR2B	0.582
FLT3	0.578
MUC1	0.583
TNFRSF11A	0.583
ROCK2	0.589
JAK1	0.595
